# Hydatid Disease of the Pelvis: A Rare Manifestation With Diagnostic Challenges—A Case Report and Comprehensive Literature Review

**DOI:** 10.1002/ccr3.70931

**Published:** 2025-09-20

**Authors:** Saurav Jha, Matsendra Jha, Sweta Singh, Sulav Kumar Jha, Bistrit Dahal, Anamika Adhikari, Sanskriti Dev

**Affiliations:** ^1^ Department of Radiology Patan Academy of Health Sciences Kathmandu Nepal; ^2^ Department of Surgery Manipal Teaching Hospital Pokhara Nepal; ^3^ B.P Koirala Institute of Health Sciences Dharan Nepal; ^4^ Manipal College of Medical Sciences Pokhara Nepal

**Keywords:** case report, echinococcus, hydatid disease, parasite

## Abstract

Hydatid disease, a parasitic infection caused by a tapeworm of the genus *Echinococcus*, predominantly affects the liver and the lungs, but its location in the pelvis is extremely rare. Pelvic hydatid cysts can sometimes mimic as an adnexal mass and can pose a significant diagnostic challenge, with subsequent delays in the treatment. We herein aim to highlight the importance of considering hydatid disease in the differential diagnosis of a pelvic cystic lesion, especially in endemic regions, to avoid misdiagnosis and intraoperative complications, ensuring timely treatment and reduced morbidity.

## Graphical abstract

Axial CECT of abdomen and pelvis showing multiple Hydatid Cysts.

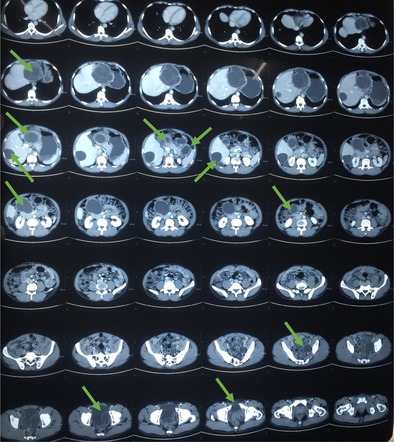

## 1 Introduction

The larval stage of the parasitic tapeworm of genus *Echinococcus* primarily affects the liver, lungs, and is classified as a zoonotic infection. Its occurrence in the pelvic cavity is rare, accounting for only a few reported cases. The pelvic cavity, due to its limited and rigid space, is an uncommon site for cyst localization. However, when affected, the cyst can exert pressure on adjacent pelvic structures, particularly the urinary bladder and rectum [1].

Humans are accidental intermediate hosts, typically acquiring infection through ingestion of food or water contaminated with dog feces. The ingested eggs, upon reaching the intestine, release embryos that penetrate the intestinal wall and disseminate hematogenously, most commonly to the liver. Pelvic hydatid cyst is believed to occur secondarily through hematogenous or lymphatic spread [2]. Therefore, the patient's occupational history, such as animal husbandry, and the level of sanitation become important risk factors.

Cystic echinococcosis may remain asymptomatic for years, with symptoms appearing due to cyst expansion, pressure effects on surrounding organs, or complications like rupture [3]. Recurrence after surgery often results from either intraoperative spillage of viable scolices or incomplete removal of the germinal layer. Diagnostic imaging modalities, including ultrasound, Contrast‐Enhanced CT (CECT), and MRI, play a vital role in detecting these cysts, while serological tests assist in confirming the diagnosis [4].

Here, we present a rare case of recurrent hepatic and pelvic cystic echinococcosis in a 62‐year‐old Nepali male, four years post‐laparotomy. This case highlights the complexity of managing multi‐organ hydatid disease and emphasizes the need for long‐term monitoring in endemic regions. We outline the clinical features, diagnostic approach, and surgical management of the patient, underscoring the importance of considering hydatid cysts as a differential diagnosis of pelvic masses, especially in endemic regions.

## 2 Case History/Examinations

A 62‐year‐old male presented with chief complaints of lower abdominal pain and swelling over the epigastric, umbilical region, and hypogastric region for the past 2 months. The patient reported a gradual onset of lower abdominal discomfort associated with progressively increasing swelling in the hypogastric region. On further inquiry, the patient also complained of increased frequency of micturition but denied a history of vomiting, diarrhea, significant weight loss, fever, and jaundice. He had a similar complaint 4 years ago, for which he underwent laparotomy and surgical excision of a hepatic hydatid cyst.

The patient was a retired farmer by occupation with a history of close contact with livestock (sheep) and domestic dogs. Additionally, on questioning his diet, it was revealed that it mostly consisted of mixed food and had a habit of consuming locally sourced spring water. There was no history of chronic illnesses such as hypertension, diabetes, and tuberculosis, with no history of smoking and alcoholism. Family history was noncontributory, with no similar illness noticed among close relatives and he was not under any long‐term medications.

On general examination, he was conscious, oriented, afebrile, and vitally stable. There were no signs of anemia, jaundice, cyanosis, clubbing, or lymphadenopathy. Per‐abdominal examination revealed a healed midline surgical scar extending from the xiphoid process to the symphysis pubis. Additionally, palpable lumps were noted in the supra‐umbilical and epigastric regions, both showing a positive cough impulse, suggestive of incisional hernia. The rest of the systemic examination, including cardiovascular, respiratory, and neurological systems, was normal.

## 3 Differential Diagnosis/Investigations/Treatment

**FIGURE 1:**
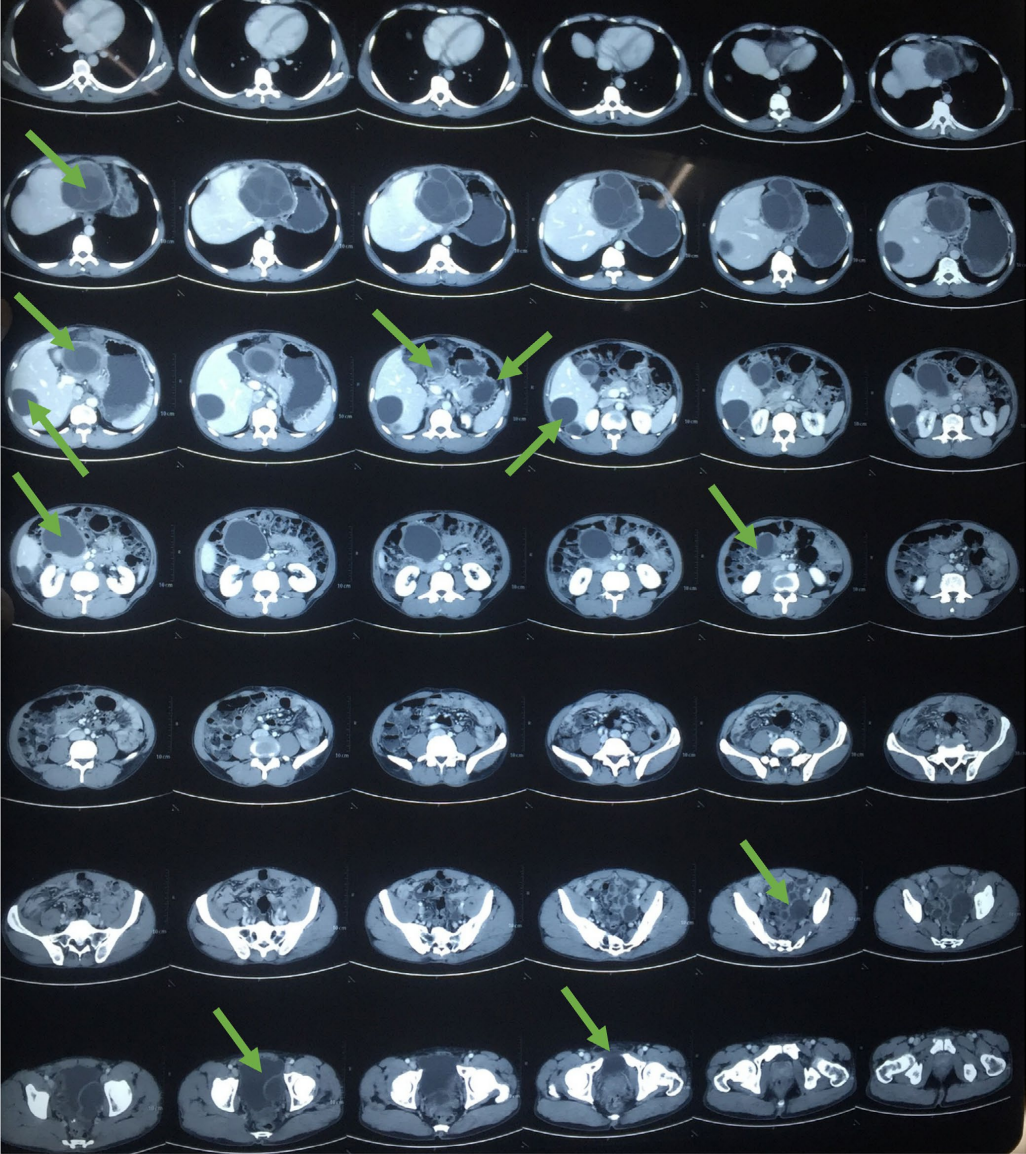
Axial CECT abdomen and pelvis reveals multiple hydatid cyst in liver, subhepatic region, lower abdomen and pelvis (arrows).

**FIGURE 2:**
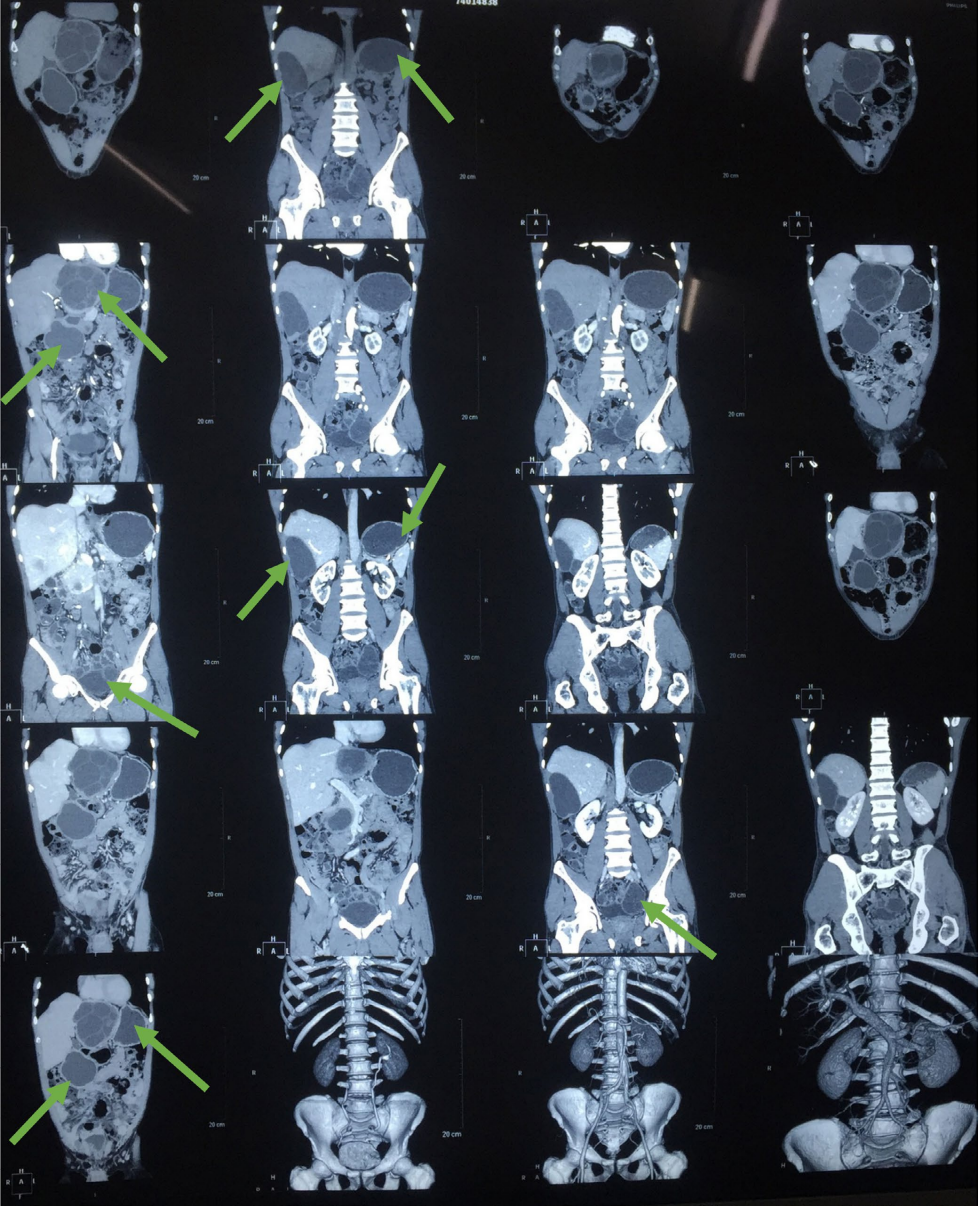
Coronal CECT abdomen and pelvis reveals hydatid cyst in the pelvic region abutting the urinary bladder with hydatid cyst in the subhepatic region and liver (arrows).

Laboratory investigations including complete blood count, urine routine examination, liver and renal function tests were all within normal range. All these clinical features, history, and lab investigations led us to the list of differential diagnosis such as neoplasm, urachal cyst, mesenteric cyst, lymphocele, pelvic abscess, and pelvic hydatid cyst. There was no significant history of weight loss, which helped us to rule out neoplasm; the age of the patient was a supporting factor in ruling out the diagnosis such as urachal and mesenteric cyst, and the absence of fever excluded pelvic abscess. Pertaining to his history of hydatid cyst, echinococcus (hydatid serology) IgG was also performed and found to be markedly elevated. An ultrasonography (USG) of the abdomen and pelvis revealed a multiseptated cystic lesion measuring 6.4 × 4.4 cm in the right lobe of the liver, and a larger cystic lesion measuring 7 × 4.5 cm was also noted in the pelvis. To further delineate the anatomy and extent, a CECT scan of the abdomen and pelvis was performed, which confirmed the presence of cystic lesions in both the right and left subhepatic regions and in the pelvis, the latter abutting the urinary bladder (Figures 1 and 2).

## 4 Outcome and Follow‐Up

**FIGURE 3:**
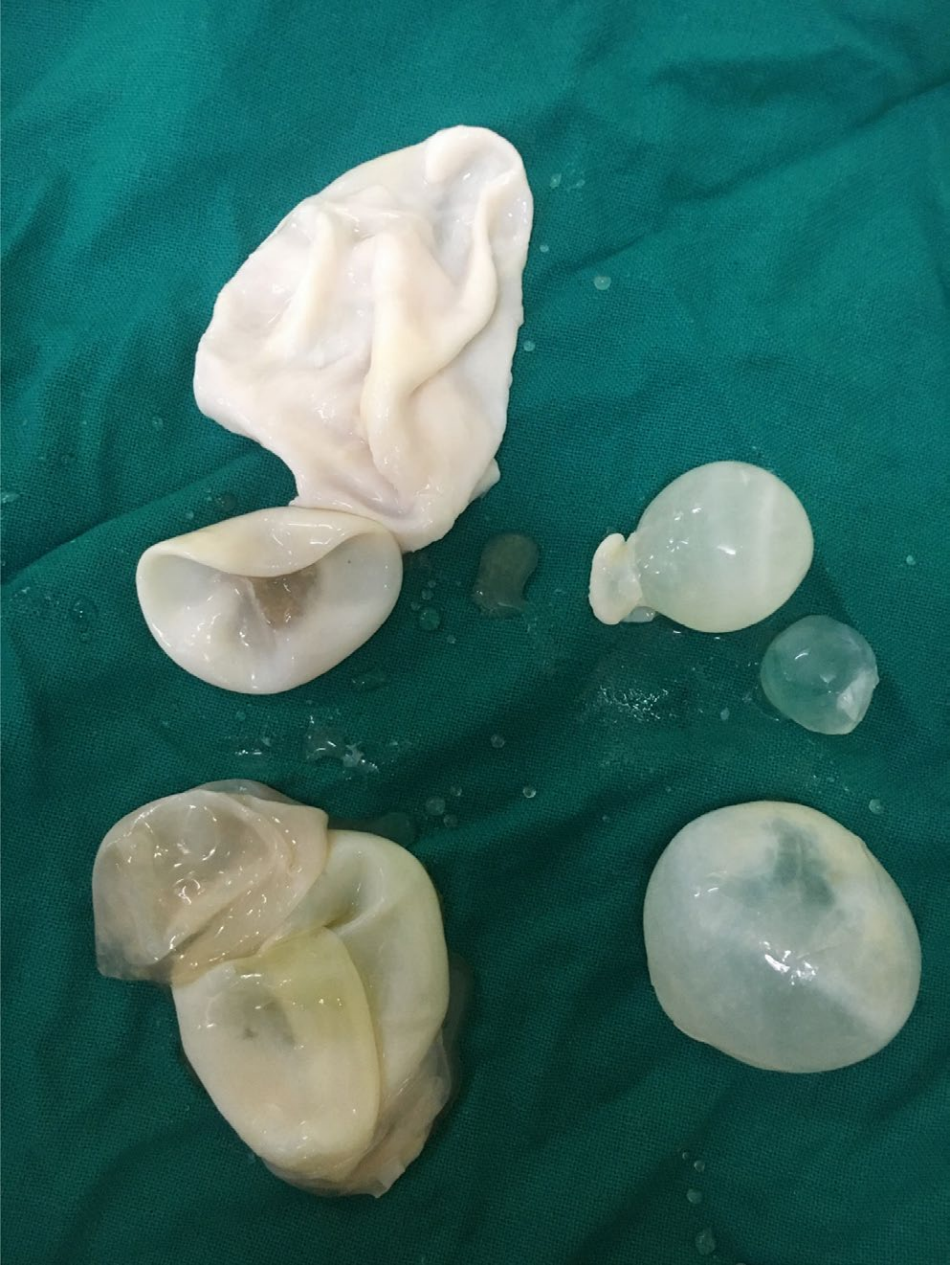
Post‐surgery picture revealing multiple hydatid cysts extracted from the pelvis and liver.

Given the radiological findings and past surgical history, a diagnosis of recurrent hepatic and pelvic hydatid disease with incisional hernias was made. The case was discussed with the surgical team, and the patient underwent laparotomy with partial pericystectomy of pelvic hydatid cyst, deroofing of hepatic hydatid cyst, and PAIR (Puncture, Aspiration, Injection, and Reaspiration) technique for hydatid cyst management (Figure 3). Peritoneal lavage and drainage were carried out, and primary repair of the incisional hernias was completed during the same procedure.

Postoperatively, the patient was started on albendazole 400 mg twice daily for 3 months to minimize the risk of recurrence. He was advised regular follow‐up with serial imaging studies and monitoring liver function. The patient was counseled regarding personal hygiene, the importance of deworming domestic animals, and the need for using safe water to prevent the further recurrence of episodes of echinococcosis. His recent follow‐up was uneventful.

## 5 Discussion

Hydatid cyst disease is among the few of zoonotic diseases endemic to the Middle East, South America, East Africa, and the Mediterranean countries [5]. Echinococcosis continues to be a significant public health concern in the countries like Nepal, and studies conducted on dogs have shown that the incidence and prevalence of Echinococcus infections are notably higher in areas where livestock farming and animal slaughter are the primary means of livelihood [6].

Echinococcus species, typically residing in the intestines of dogs and other canines, gets entry to the intestines of intermediate hosts via the ingestion of food and water contaminated with the parasitic eggs. Humans, although considered accidental intermediate hosts, may acquire the infection due to close handling of infected dogs or drinking contaminated water [7]. Upon ingestion, the eggs hatch in the small intestine and the larvae gain access to portal circulation. Furthermore, it passes to the liver, which acts as the primary filter, trapping the majority of larva, while a small proportion bypasses it and gets lodged in the lungs, the secondary filter [8]. As a result, the cysts are most often found to be localized in the liver (68.8%–74.8%) and lungs (17.2%–24%) [3]. In contrast, localization of hydatid cysts in pelvic organs, particularly in males, is exceedingly rare and is thought to occur via secondary dissemination from lymphatic and hematogenous routes, with a reported frequency of 0.2%–0.9% in selected locations [9].

The clinical symptomatology of hydatid disease depends on the size, the site, and the extent of the lesion. Additionally, the patient may be asymptomatic, and cysts are discovered incidentally as a palpable mass, voiding difficulties, and pelvic pain [3]. Also, eosinophilia is a common response to parasitic infection, especially helminths, but cystic echinococcus is not often recognized as a frequent cause of eosinophilia [10]. A pelvic hydatid cyst, due to its presence in a confined anatomical space, can exert pressure on adjacent structures, and the urinary bladder is the most affected structure, followed by the rectum [1].

The diagnosis of cystic Echinococcus primarily relies on the detection of cystic lesions through imaging techniques such as CECT, Ultrasonography (USG), and X‐rays, with confirmation typically achieved through serological testing for specific anti‐echinococcal antibodies [4]. Ultrasound is the imaging modality of choice due to its availability, lack of radiation, and high resolution for diagnosis, staging, differential diagnosis, and follow‐up of most abdominal cystic lesions, with an established role in interventional treatment of cystic echinococcosis (CE) [11]. Alongside the Gharbi classification, the WHO Informal Working Group on Echinococcosis (WHO‐IWGE) classification—which closely resembles it—is also commonly used for interpreting ultrasound images [12]. WHO‐IWGE classification of hydatid cyst stages into three clinical groups:

- The “active” group includes developing cysts, which may be unilocular (CE1) or multivesicular with daughter vesicles (CE2) and which are usually found to be viable.
- The “transitional” group (CE3) includes both cysts with detachment of endocyst (CE3a) and predominantly solid cysts with daughter vesicles (CE3b),
- The “inactive” group (CE4 and CE5) exhibits involution and solidification of cyst content with increasing degrees of calcification and is nearly always found to be nonviable.

This WHO classification provides a rational basis for choosing an appropriate Cystic Echinococcosis treatment scheme and follow‐up, that is, surgery, percutaneous treatment (PAIR), benzimidazole chemotherapy, or simply “watch & wait.” The WHO classification divides CE3 hydatid cysts into two distinct morphological subtypes: CE3a and CE3b. CE3a is characterized by floating membranes, often referred to as the “water‐lily sign,” while CE3b typically appears as a mostly solid cyst containing daughter vesicles. This subdivision is based on differences in both imaging appearance and therapeutic response—CE3a cysts generally respond well to PAIR and albendazole, whereas CE3b cysts tend to show a poor response to these treatments [13]. CT remains crucial for identifying complications, assessing involvement of lungs and bones, planning surgery, diagnosing recurrences, and clarifying uncertain ultrasound findings. Unenhanced CT is ideal for detecting calcifications, which can appear in all stages of CE, not just active ones [14]. Additionally, CECT plays a key role in differentiating CE from other focal liver lesions [13]. In cases where antibody levels are undetectable, image‐guided fine needle aspiration, particularly under ultrasonographic guidance, has recently been employed as a diagnostic tool in uncertain cases [15].

Management of hydatid cysts involves a multimodal approach that includes surgical intervention (radical or conservative), PAIR (Puncture‐Aspiration‐Injection‐Reaspiration), antiparasitic therapy, and active surveillance in selected cases. Surgical treatment remains the mainstay for hepatic and extrahepatic hydatid cysts, especially in complicated and large cysts or those compressing adjacent structures [16]. PAIR is a minimally invasive alternative indicated for inoperable patients, postsurgical recurrence, or when the patient declines surgery [17]. Although recurrence in PAIR techniques is high, these methods are widely adopted because they avoid open surgery, can be performed under local anesthesia or sedation, reduce hospital stay, and promote faster recovery. However, this is contraindicated in cases where cysts have fistulized into the bile duct. The surgical treatment of hydatid cyst disease has been associated with an overall recurrence rate of approximately 10% and can be reduced with the help of drugs [18]. Medical therapy using albendazole or mebendazole is recommended, and combination therapy with praziquantel may enhance treatment efficacy due to its protoscolicidal properties [16]. Albendazole is favored for treatment due to its superior intestinal absorption and its ability to reach a higher concentration within cysts [19]. Additionally, a treatment duration of at least three months is associated with favorable outcomes, including marked reduction in cyst size and greater likelihood of complete recovery [20].

Recent literature, including the study by Rahdar et al., has demonstrated remarkable efficacy in the prophylaxis (100%) and treatment (97%) of experimental hydatid cysts using a combination of albendazole, interleukin‐12 (IL‐12), and interferon‐gamma (IFN‐γ) [21]. Furthermore, advancements in nanotechnology have been pivotal in addressing longstanding challenges associated with conventional chemotherapy for hydatid disease—particularly anatomical barriers, cyst wall, and deep‐seated localization—which often render traditional ways of treatment suboptimal and dose escalation potentially hazardous. Nanotechnology offers a transformative approach to drug delivery, with nanocarriers and encapsulated formulations significantly enhancing the solubility, stability, and bioavailability of therapeutic agents [22]. These nanostructured systems enable more targeted, precise, and effective delivery of chemotherapeutics directly to the cystic lesions, potentially revolutionizing current treatment paradigms [22].

Usually, hydatid cysts are localized in the liver and the lungs; still, in our patient, cysts were unusually located in the pelvis. With this case report, we would like to highlight the rarity of pelvic hydatid cysts as an uncommon manifestation of echinococcus that contrasts with its typical hepatic and pulmonary involvement. Notably, we present clinical features, diagnostic challenges, and imaging findings associated with this unusual location. Furthermore, we emphasize the need to consider hydatid disease in the differential diagnosis of pelvic masses, especially in endemic regions where such cases could be overlooked. Additionally, this report outlines the management approach, encompassing surgical excision and adjunctive chemotherapy. Ultimately, by documenting this unusual presentation, this report aims to enhance clinical awareness and support early diagnosis and effective management of hydatid cysts in an uncommon anatomical location.

## 6 Conclusion

Pelvic hydatid cyst is among the rarest manifestations of *Echinococcus granulosus* infection and should be considered in the differential diagnosis of cystic pelvic masses, particularly in patients from endemic regions complaining of nonspecific lower abdominal or pelvic pain. Through careful clinical evaluation and appropriate radiological investigations, we successfully diagnosed a hydatid cyst localized to the male pelvis—an exceptionally uncommon site of involvement. While abdominal ultrasound may aid in the initial evaluation, CECT scan remains the imaging modality of choice for both diagnosis and surgical planning. In this report, we emphasize the rarity of pelvic hydatid cyst and highlight the significance of considering hydatid etiology in atypical pelvic presentation. With this case, we aim to enrich the literature with clinical insights into this rare anatomical localization of hydatid disease.

## Author Contributions

**Saurav Jha:** conceptualization, supervision. **Matsendra Jha:** conceptualization. **Sweta Singh:** supervision, writing – review and editing. **Sulav Kumar Jha:** conceptualization, data curation, writing – original draft. **Bistrit Dahal:** writing – review and editing. **Anamika Adhikari:** writing – review and editing. **Sanskriti Dev:** writing – review and editing.

## Consent

Written informed consent was obtained from the patient to publish this report in accordance with the journal's patient consent policy.

## Conflicts of Interest

The authors declare no conflicts of interest.

## Data Availability

Data will be provided by the corresponding author upon reasonable request. Images will be uploaded in separate files.
